# Microstructure Evaluation and Impurities in La Containing Silicon Oxynitrides

**DOI:** 10.3390/nano11081896

**Published:** 2021-07-23

**Authors:** Abbas Saeed Hakeem, Sharafat Ali, Thomas Höche, Qasem Ahmed Drmosh, Amir Azam Khan, Bo Jonson

**Affiliations:** 1 Interdisciplinary Research Center for Hydrogen & Energy Storage (IRC-HES) Research Institute, King Fahd University of Petroleum & Minerals, Dhahran 31261, Saudi Arabia; ashakeem@kfupm.edu.sa (A.S.H.); drmosh@kfupm.edu.sa (Q.A.D.); 2 Department of Built Environment and Energy Technology, Linnæus University, SE-351 95 Växjö, Sweden; bo.jonson@lnu.se; 3 Leibniz Institute of Surface Engineering IOM, Permoserstr. 15, D-04318 Leipzig, Germany; thomas.hoeche@imws.fraunhofer.de; 4 Fraunhofer Institute for Microstructure of Materials and Systems IMWS, Walter-Huelse-Strasse 1, D-06120 Halle, Germany; 5 School of Chemical and Materials Engineering, National University of Science & Technology, Islamabad H-12, Pakistan; amirazamkhan@msn.com

**Keywords:** oxynitride glass, nitrogen enrich, transparency, defects, electron microscopy, characterisation

## Abstract

Oxynitride glasses are not yet commercialised primarily due to the impurities present in the network of these glasses. In this work, we investigated the microstructure and instinctive defects in nitrogen rich La−Si−O−N glasses. Glasses were prepared by heating a powder mixture of pure La metal, Si_3_N_4_, and SiO_2_ in a nitrogen atmosphere at 1650–1800 °C. The microstructure and impurities in the glasses were examined by optical microscopy, scanning electron microscopy, atomic force microscopy, and transmission electron microscopy in conjunction with electron energy-loss spectroscopy. Analyses showed that the glasses contain a small amount of spherical metal silicide particles, mostly amorphous or poorly crystalline, and having sizes typically ranging from 1 µm and less. The amount of silicide was estimated to be less than 2 vol. %. There was no systematic relation between silicide formation and glass composition or preparation temperature. The microstructure examination revealed that the opacity of these nitrogen rich glasses is due to the elemental Si arise from the decomposition reaction of silicon nitride and silicon oxide, at a high temperature above ~1600 °C and from the metallic silicide particles formed by the reduction of silicon oxide and silicon nitride at an early stage of reaction to form a silicide intermetallic with the La metal.

## 1. Introduction

Silicon-based oxynitride glasses have become a well-established technological process in glass science and technology since their introduction in the late 1960s. Studies have shown that nitrogen incorporation induces significant structural rearrangements within the glass network, leading to superior thermal, mechanical, optical, and chemical properties. The structural role of nitrogen in the silicate glass network is similar to oxygen, except that some of the nitrogen is linked with three silicon tetrahedra. Most of the potential applications have not been commercialised yet primarily because of the challenges remaining with scaling up the process to mass production and developing a more cost-efficient synthesis method. Another significant challenge is still the production of optically transparent glasses in the visible region. In this paper, we report on the possible reason for the non-transparency of these glasses.

The synthesis of metal containing SiON and SiAlON glasses involve the reaction of solid Si_3_N_4_, SiO_2_, (AlN/Al_2_O_3_), and metal oxides, yielding glasses with nitrogen contents up to typically ~30 e/o, or by reacting NH_3_ or N_2_ with molten oxides, yielding glasses with nitrogen content less than 10 e/o. Studies have shown that using metal or metal hydride as starting materials, glasses can be synthesised in the M−Si−O−N (where M is metal) systems [1,2,3,4,5,6,7,8,9,10]. By employing a pure metal or metal hydride, glasses can be obtained with broad compositional ranges retaining high amounts of nitrogen and modifiers.

As compared to oxide glasses, oxynitride glasses are translucent in the visible range. Often these glasses have a grey to black appearance, depending on the concentration of nitrogen and modifier in the silicate glass network. The intensity of the opaqueness is not incessantly attributed to high nitrogen content in the glass network [11,12]. The transparency issue of oxynitride glasses has been investigated [13,14,15,16], but with no significant progress toward obtaining nitrogen-enriched glasses with fully transparency [17,18,19]. One of the most common reasons for the opacity of the oxynitride glasses is the alleged precipitation of silicon [13,16,20], which is due to the decomposition of Si_3_N_4_ [21] at a temperature above 1600 °C. Meanwhile, avoiding decomposition of Si_3_N_4_ by applying low synthesis temperature, e.g., 1450 °C, high transparent glasses have been obtained for some compositions. Silicon nitride ceramics also contain free silicon, generally, the higher the amount of Si, the darker the glasses/ceramics, and the less transparent glasses/ceramics are. Sharafat et al. [5,22] reported that the Ca-containing SiON system reveals better transparency for the glasses containing high nitrogen contents. This is attributed to the use of high purity precursors and slow cooling rates resulting in improved transparency. Furthermore, it was found that the substituting of Ca with Mg in the Ca−Si-O-N system enhances the transparency of the glasses. In contrast, silicon-free oxynitride glasses, e.g., P−O−N-based, and oxynitride glasses prepared via sol–gel methods are transparent. Recently, Sharafat et al. [23,24,25] have reported amorphous transparent thin films in the nitrogen enrich Mg/Ca−Si−O−N systems prepared by reactive RF magnetron co-sputtering from Mg/Ca and Si targets in an N_2_/O_2_/Ar gas mixture. These thin films are free from elemental silicon and metallic silicides impurities, as found in bulk Mg/Ca−Si−O−N systems and contain an unprecedented amount of modifier and nitrogen. Furthermore, thin films in the Mg/Ca−Si−O−N systems have higher hardness, elastic moduli, and refractive index as compared to bulk glasses in the said systems of similar compositions.

Considering the growing interest in oxynitride glasses, the objective of this work is to provide information and formulate a reasonably complete and consistent description of microstructure and impurities in the nitrogen rich glasses in the La−Si−O−N system.

## 2. Experimental

Preparation of La−Si−O−N glasses was carried out using powder mixtures of La metal (ChemPure, 99.9%, Karlsruhe, Germany), Si_3_N_4_ (UBE, SNE10, Tokyo, Japan), and SiO_2_ (Aerosil 50, Hanau-Wolfgang, Geramny). Ten-gram batches of each composition were ground in a glovebox compartment under Ar atmosphere to avoid the mixture from oxidation. The mixtures were melted in niobium crucibles at 1650–1800 °C, in a nitrogen atmosphere, using a radio frequency furnace. Further details on synthesis condition, composition, and characterisations are given in [1,2].

The obtained glasses were examined by X-ray powder diffraction (XRPD), using a Panalytical X’pert PRO MPD (Almelo, Netherlands) diffractometer and CuK_α1_ radiation. Samples were analysed by light microscope and scanning electron microscopy to observe the surface morphology. The microstructure examination was carried out using a Jeol (JSM 7000F, Tokyo, Japan) scanning electron microscope. Micrographs of the specimens were acquired in backscattered electron mode (BSE) to obtain compositional contrast. The cationic percentage compositions of glass and crystalline phases were evaluated through EDX point analyses, on polished and carbon-coated surfaces, ten (10) analyses on each sample, with a Si detector and a LINK INCA (Oxford Inc., High Wycombe, UK.) program system. N_2_ and O_2_ contents were determined by using combustion analysis with a Leco Detector (TV-436DR, Leoben, Austria) equipment. Raman spectroscopy measurement was performed with a dispersive confocal Raman microscope (Renishaw inVia, New Mills, UK.) using 633 nm laser excitation lines. The spectra were obtained in the range from 200 to 2200 cm^−1^ with a resolution of 2 cm^−1^, the sample spot size of the Raman microscope is ~0.5 µm in diameter. The surface topography of the glasses was characterised by atomic force microscopy (AFM) (Bruker, NanoScope Controller, Santa Barbara, CA, USA) in tapping mode for both height and phase image using an RTESPA300° probe. The analysis was performed on the freshly fracture surface of glass samples. The microstructure of the glass and silicide morphology were investigated by transmission electron microscopy (TEM) and electron energy-loss spectroscopy (EELS). For TEM observations, the bulk sample was plane-parallel ground, one-sided dimpled and polished to ~20 µm residual thickness, Argon ion beam polished (2.5 kV, 4° angle of glancing incidence), and selectively carbon coated [26]. Transmission electron microscopy (TEM) studies were carried out using a Hitachi TEM (H-8100 operated at 200 keV, equipped with EDXS, Krefeld, Germany) and a JEOL TEM (JEM 4010 operated at 400 keV). EELS analyses were performed using a dedicated scanning transmission electron microscope (VG HB501 UX, Tokyo, Japan) equipped with a cold field emission gun and a high-resolution electron energy-loss spectrometer (Gatan Enfina 1000, Pleasanton, CA, USA).

## 3. Results and Discussion

### 3.1. Influence of Time and Temperature

The glass-forming region and properties of La−Si−O−N glasses were reported by Hakeem et al. [1,2]. These glasses were prepared by incorporating the La metal in the reaction mixture rather than using La_2_O_3_—as typically used—with silicon nitride as the nitrogen source. The obtained glasses contained up to 62 e/o of La and 68 e/o of N, and showed a larger glass-forming region than previously reported [27,28,29,30,31]. The effect of time and temperature are, to a certain extent, interrelated. By increasing the temperature, the melting process speeds up and leads to high weight loss. The amounts of silicide did not show apparent variation with glass composition or preparation temperature.

### 3.2. Influence of Raw Materials and Nitrogen Pressure

All of the glass samples were prepared from a similar source of powders of La, SiO_2,_ and Si_3_N_4_ and using identical nitrogen pressure, i.e., one (1) atmospheric pressure. Therefore, it is difficult to ascertain the effects of raw materials and nitrogen pressure in the present study. However, Korgul et al. [12] concluded that the transparency of the glasses improved by using high-purity quartz powder instead of precipitated silica. Furthermore, it was observed that the substitution of Si_3_N_4_ by AlN (in the SiAlON system) and increasing nitrogen pressure show no distinction improvement in the transparency. Sharafat et al. [4] reported glasses in the Sr−Si−O−N system turn more transparent by using SrD_2_ rather than Sr metal as a modifier. Furthermore, the glasses made by using SrD_2_ contain much smaller amounts of Sr silicides as compared to the glasses prepared by Sr metal. Pasto et al. [32] reported that the origin of the impurities was in the raw materials; additionally, impurities were picked up during powder processing and melting. Iron is probably the most common impurity found in the batch material for glass (impurity from the precursors) and can readily oxidise silicon nitride at quite low temperatures to form FeSi_2_. Furthermore, decomposition was not noticeably diminished when using high-purity Si_3_N_4_ as a batch component. As mentioned earlier that no iron impurities were present in the used raw materials for the preparation of nitrogen-enriched glasses in the La−Si−O−N system, both SEM and TEM investigation endorse no existence of iron silicide in the present glasses.

### 3.3. Observation of Interface between Glass and Niobium Crucible

**Figure 1:**
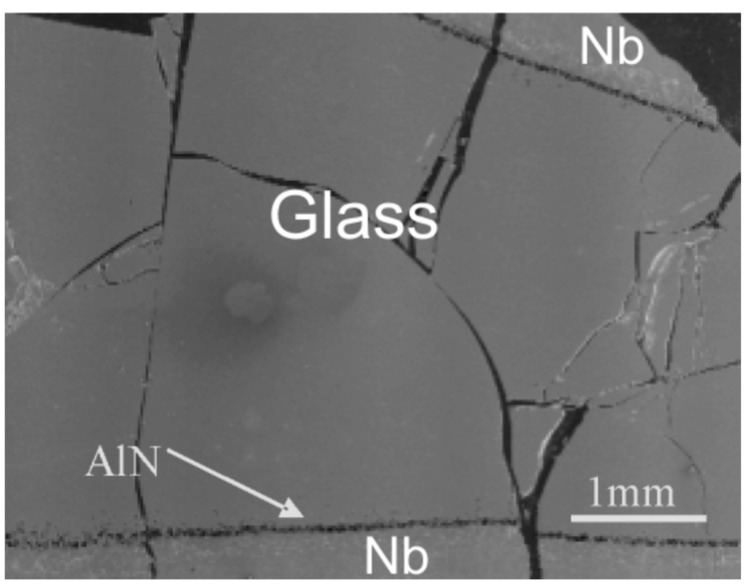
SEM image La−Al−Si−O−N glass prepared in the Nb crucible and AlN as a starting powder, the arrow indicating the wall of the Nb crucible [9].

All the glasses were prepared by standard procedures, as described in the experimental section. It was observed that there is no reaction between La−Si−O−N melt and niobium crucible used in the study, although the melt did adhere to the surface of the crucible. Similarly, no reaction between the melt and Nb crucible was observed in other AE−Si−O−N (AE = Mg,Ca,Sr,Ba) [3,4,22,33] systems prepared by a similar synthesis route as described for La−Si−O−N glasses. However, it was observed that the La−Si−Al−O−N glass in which AlN was added does react with the Nb crucible and some of AlN remained undissolved in the liquid because of its strong bonding and stability of the AlN and perhaps much longer holding time required for AlN to dissolve into the liquid phase. Therefore, aluminium had to be added in oxide (and perhaps in metallic) form, i.e., as Al_2_O_3_ instead of AlN, as an admixture to the composition. Experimental observation indicates that Al_2_O_3_ reacts and dissolves into the liquid much faster than AlN. Figure 1 reveals some undissolved AlN near the crucible wall. It is thus easier to dissolve Al_2_O_3_ than AlN in the La-Si-Al-O-N system. Recently, Natalia et al. [34] reported the dissolution of Nb metal from Nb crucible in the phosphorus-based oxynitride glasses. The addition of Al_2_O_3_ into the La−Si−O−N system showed that the glasses can be prepared at lower temperature and in a shorter time. Furthermore, the addition of Al to the La−Si−O−N system enhances transparency and minimises losses of components during preparation.

### 3.4. Optical Microscopy Observations

**Figure 2:**
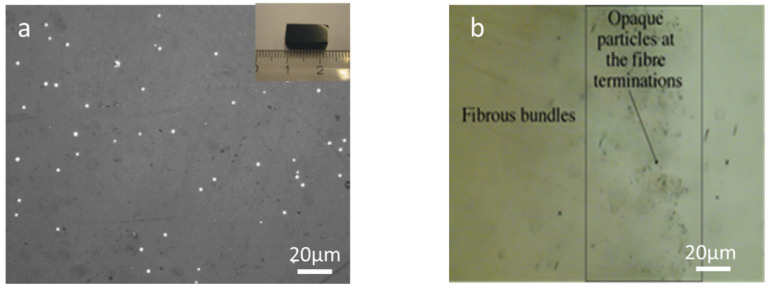
Optical micrographs were showing the presence of (**a**) La silicides (white spots) in nitrogen-enriched La−Si−O−N glass and a polished glass piece of (inset). (**b**) Fibrous bundles in La−Si−O−N glass.

Light microscopy was used to examine the surface of polished glass samples. Most of the glasses were found to be opaque and generally had a brown colouration and reflecting white spots, as shown in Figure 2a. Additionally, further experiments confirm that glasses contain spherical La silicide (LaSi_2_) particles in the matrix. The amount was estimated in the range of around 2 to 4 vol. % and varied upon the starting composition. There was no elemental silicon observed by the light microscope and SEM analysis. Figure 2b shows the character of the distribution of opaque grains. The glass samples contain a very high frequency of equidimensional rounded opaque grains reaching sizes of a few microns, although most of them are much smaller. The opaque grains are unevenly distributed, with larger, more separated grains in the more transparent parts of the glass. In contrast, dusty impregnations of microscopic opaque particles occur in the dark-coloured parts of the glass.

### 3.5. Scanning Electron Microscopy

**Figure 3:**
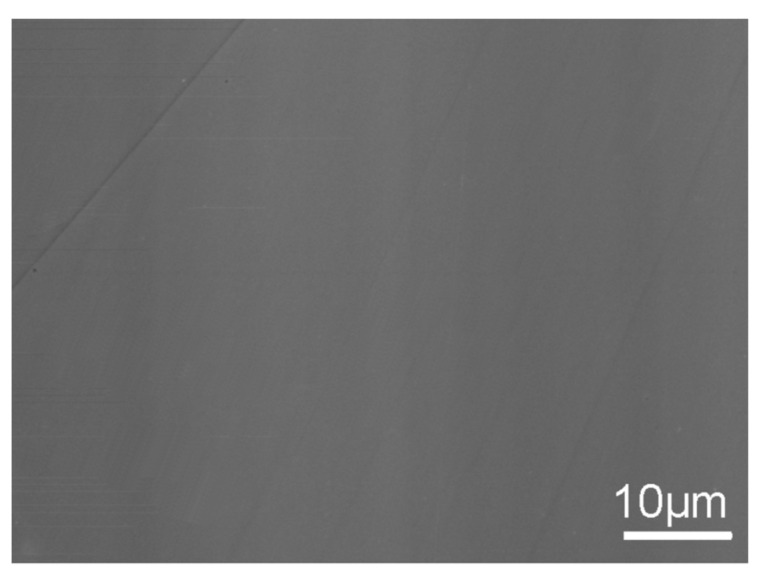
SEM image of a homogeneous glass surface with no apparent impurities [9].

SEM observations showed that glasses have homogeneous microstructures, indicating a lack of substructural features, as shown in Figure 3. However, the glasses do contain small amounts of La silicide (mostly spherical particles), as noticed by the optical microscopic analysis as well. The La−Si interaction is complex. It might occur during synthesis, as part of the initial Si_3_N_4_ and SiO_2_ is reduced at an early stage to form a silicide with La metal. This might be ascribed to one of the following two mechanisms: (a) the direct reaction between La metal and Si_3_N_4_ to produce lanthanum silicide (La_x_Si_y_) and nitrogen or (b) the reaction of lanthanum nitride + silicon, with a subsequent reaction of La and Si. At high temperature and longer holding time, the La_x_Si_y_ gradually dissolves into the glass. According to the lanthanum-silicon phase diagram [35], LaSi_2_ melts at 1730 °C and La_3_Si_2_ melts at 1470 °C. Additionally, few compositions had a lower proportion of silicide precipitation, which indicates that the formation and the quantities of silicides largely depend on the synthesis route and heat treatment procedure and not much on nitrogen concentration present in the stoichiometry.

**Figure 4:**
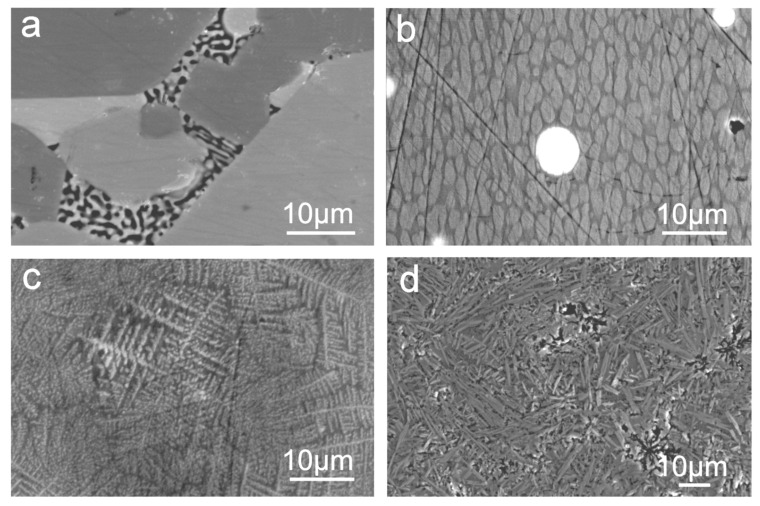
SEM images show (**a**) two phases in the La−Si−O−N glass matrix, (**b**) spherical La silicide-white, (**c**) dendritic growth in the glass and (**d**) needle- or lath-like crystalline morphologies [9].

Figure 4 shows SEM micrographs of the different morphologies and crystalline phases in the partially crystalline glass matrix. Glass sample gives weak X-ray reflections according to the XRPD pattern. However, the SEM image Figure 4a shows the presence of two glass phases by BSE compositional analysis: oxygen-rich La oxynitride phases and black coloured undissolved Si_3_N_4_. For amorphous or poorly crystallised samples, as in the La−Si−O−N system, the determination of weak X-ray reflections is complicated due to the presence of a high-intensity background (the result of inelastic scattering), leading to an underestimation of the size of crystallites. SEM image Figure 4b shows two glass phases and silicide particles (white spherical), the majority of the silicides have LaSi_2_ phase. Micrograph Figure 4c shows the growth of dendrites and Figure 4d further growth of the needle-like structure of a crystalline phase. Nitrogen-enriched La-containing SiON glasses can be described as a mixture of amorphous SiO_2_ and crystalline LaSi_2_, thus implying that phase separation occurred according to the microstructure viewpoint. Moreover, it was observed that the degree of crystallisation and the crystal sizes decrease with increasing nitrogen content. Generally, nitrogen rich glasses containing a high amount of silicides have Poisson’s ratio ranging from 25 to 36, resemble those of metallic glasses having Poisson’s ratio of ~40.

### 3.6. Raman Spectroscopy

**Figure 5:**
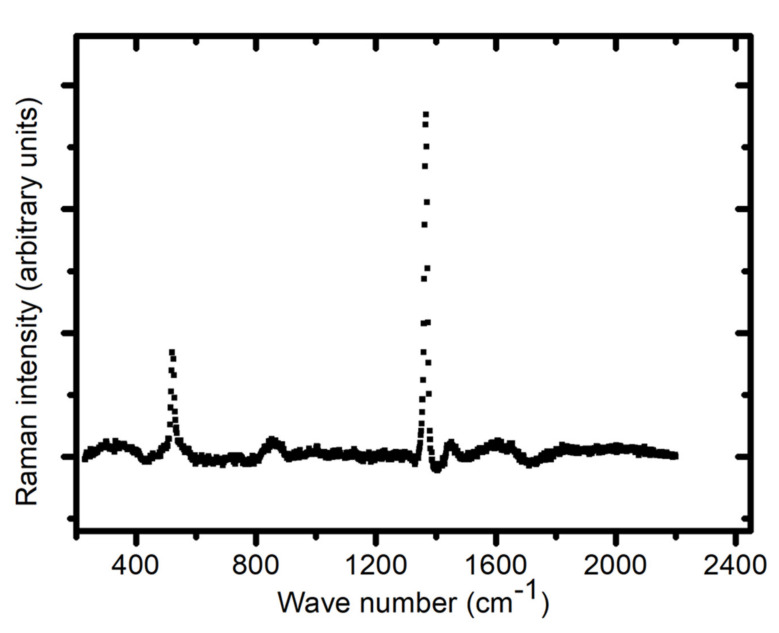
Raman spectra of silicide particles in La−Si−O−N glass.

Raman spectra show that the glasses in the La−Si−O−N system contain elemental Si and LaSi impurities. It was thus hypothesized that the absence of any signal in the XRD spectrum of these impurities is due to their size and quality effect. However, according to Raman studies, an elemental Si gives a sharp peak at 522 cm^−1^. The intensity of the Si peaks decreases with the increasing N content, and no Si peaks are observed for glasses with N contents above ~40 e/o. The spectra show a high background of fluorescence, which increases with increasing N and La contents, implying that the recorded spectra have, in general, a poorer quality for high N content glasses. Rouxel et al. [11] have reported that the strong luminescence observed in the oxynitride glasses could be due to silicon clusters of less than 7 nm in diameter. Figure 5 shows the Raman spectra of the La silicide particle.

### 3.7. Atomic Force Microscopy (AFM), Transmission Electron Microscopy (TEM) and Electron Energy-Loss Spectroscopy (EELS) Analysis

**Figure 6:**
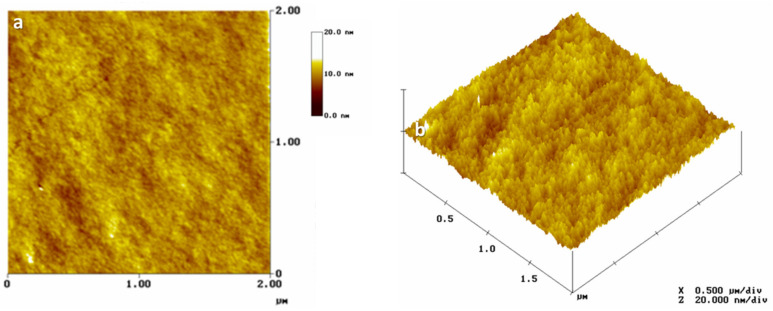
Atomic force microscopy images (**a**) 2D and (**b**) 3D, showing smooth and featureless morphology of La−Si−O−N glass.

The surface topography of La−Si−O−N glasses was investigated by AFM on the freshly fractured surfaces. The surface topography of all the studied glasses is smooth and uniform with a surface roughness value lower than 1 nm, which is close to the roughness of known silicate glasses. Figure 6 shows a uniform, defect-free and featureless structure, typical for amorphous materials with extremely fine grains.

**Figure 7:**
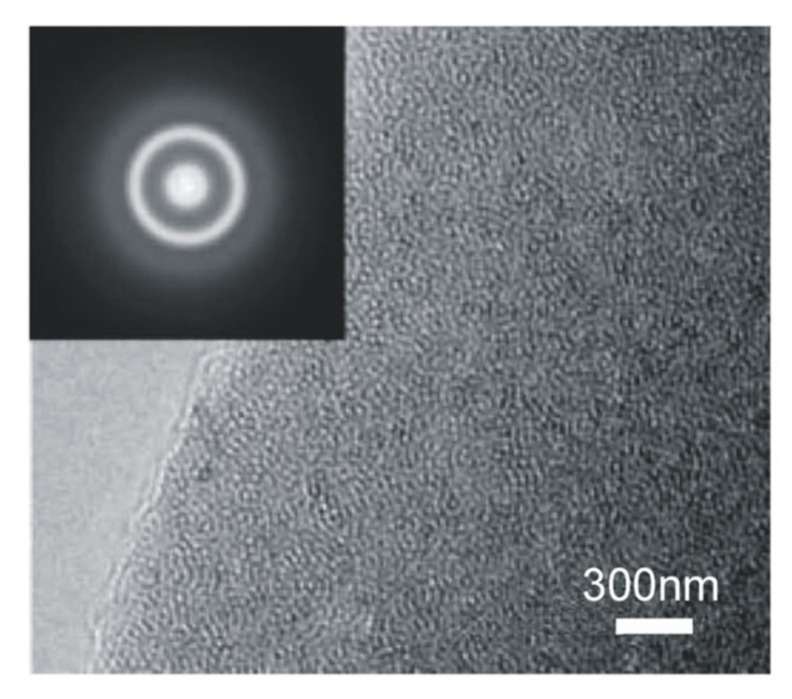
High-resolution transmission electron microscope image with the corresponding selected area diffraction pattern (inset) for La−Si−O−N glass [9].

The HRTEM images in Figure 7 show homogenous microstructure glasses with no porosity.

**Figure 8:**
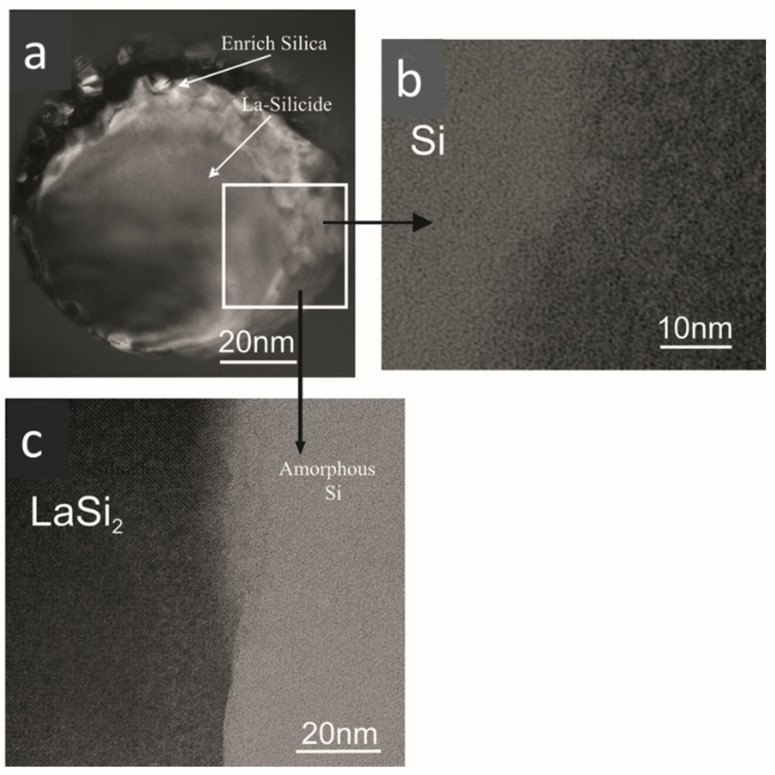
Transmission electron micrographs showing (**a**) silicide particle and (**b**,**c**) enlarged areas of Si and La-silicide, respectively [9].

**Figure 9:**
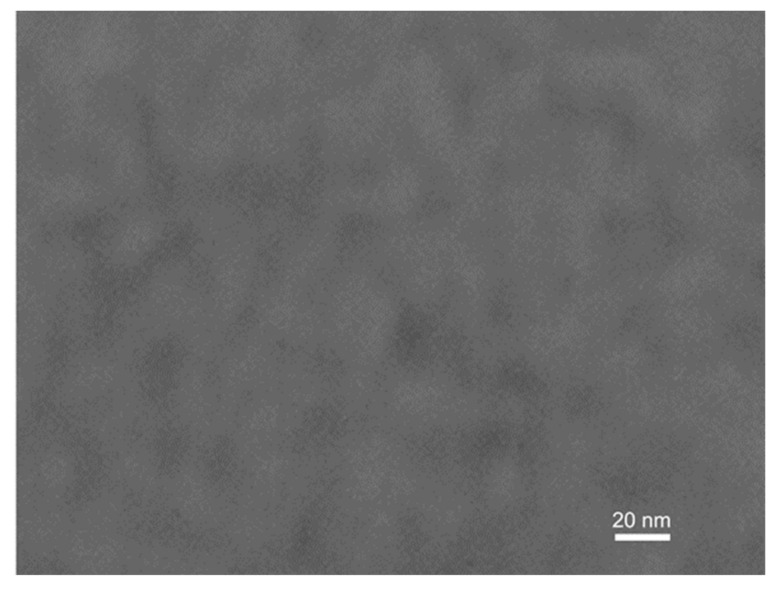
TEM image of the La−Si−O−N glass indicating some features of either phase-separation or compositional difference in the range of ~10 to 30 nm [9].

There is no evidence of substructural features in the glassy region and selected area electron diffraction (SAED) patterns confirmed the amorphous nature of the sample. Figure 8a shows the microstructure of the silicide particles of La silicide and Si-enriched particles. The majority of the La silicides are spherical and have uniform compositions. However, some of the La silicides have prominent internal structures, having entrapped particles of Si-rich phase(s), which might be Si_3_N_4_ or Si, and La enriches in the surrounding glass (Figure 8b,c). The diameter of the particles is somewhere between 1 and 5 µm, as also observed by optical microscopy investigation. These observations indicate that the lanthanum silicides steadily dissolve during the synthesis. The amounts of silicide showed no apparent variation with glass composition. However, some of the samples, as shown in Figure 9, show phase separation in the length scale of 10 to 30 nm.

The Raman spectra, as shown in Figure 5, show a sharp and intense absorption band characteristic from elemental silicon at 520 cm^−1^. The fact that no crystallinity was detected by X-ray diffraction might be due to the deficient amounts of elemental Si present in the glass matrix. Even a small amount of free silicon probably partly hinders the glass transparency, with another possible cause being the clustering or nano-phase separation evidenced by TEM, as shown in Figure 9.

**Figure 10:**
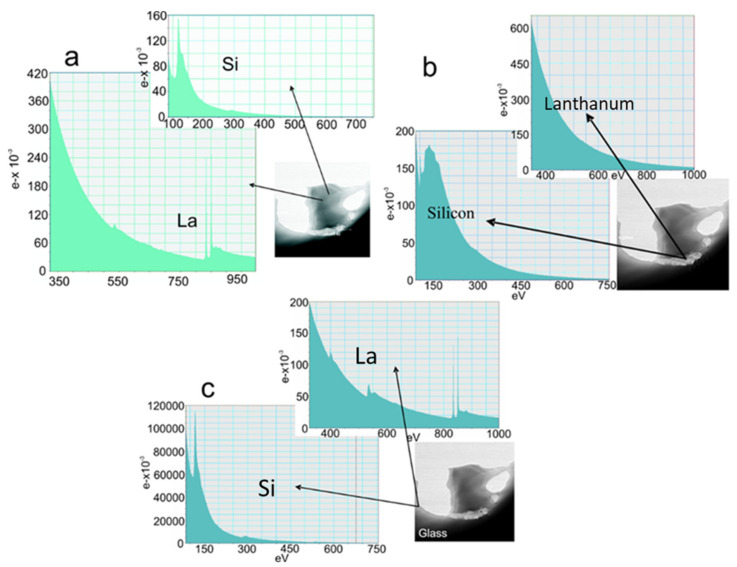
TEM (EELS analysis) images (**a**) core of the ppt, (**b**) edge of the ppt, and (**c**) edge of the glass [9].

**Figure 11:**
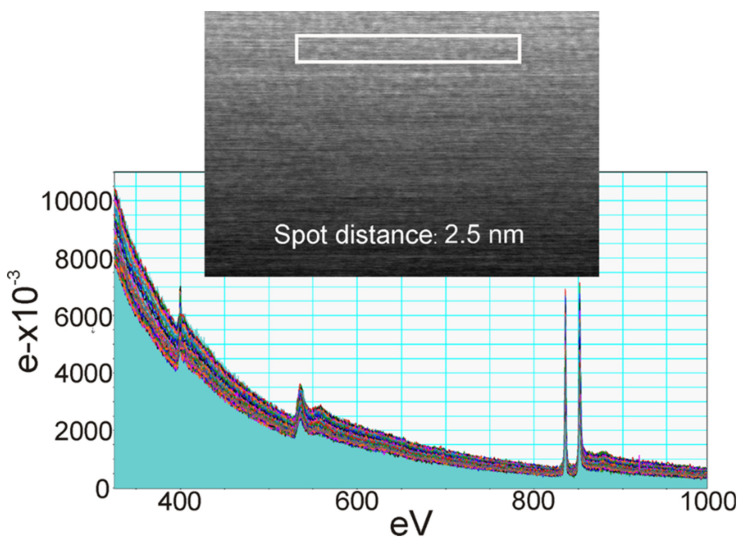
EELS spectra of compositional fluctuation along with the 100 nm line scan [9].

In Figure 10a–c, EELS spectra from a sample of La−Si−O−N glass show the presence of silicon on the edges of the precipitate. The interface between Si and glass shows no fringes indicating any ordered structure, but at the interface between Si and precipitate, ordered fringes in silicon can be seen. Figure 11 shows the EELS spectra of the amorphous region and no evidence of compositional fluctuation along with the 100 nm line scan. The size of free Si is less than 100 nm in diameter. These particles were not visible under a light microscope nor by SEM. These observations lead to the conclusion that the more nitrogen is incorporated in the glass network and the more fragile the glass becomes. Additionally, it is suggested that nitrogen favours the formation of a heterogeneous network at the nanometre scale and leads to a network structure with weak channels, resembling the ones proposed by Greaves et al. [36] and acting as a lubricant between the clusters.

### 3.8. Comparison with La−Si−O−N Thin Films

**Figure 12:**
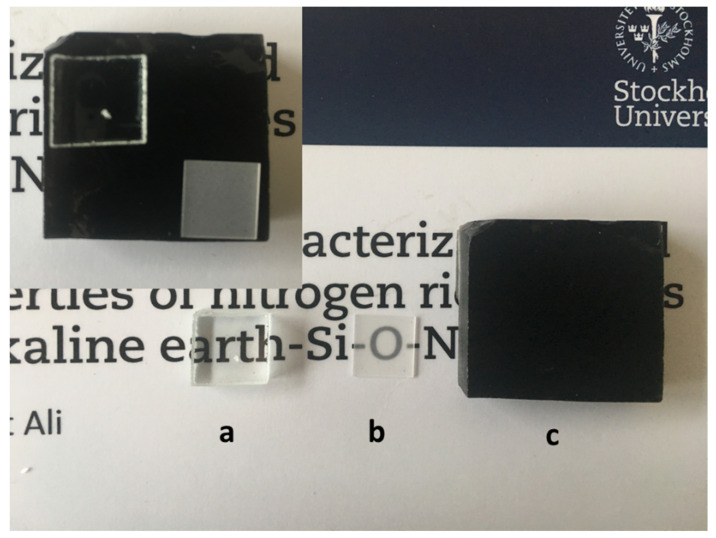
Photograph of the (**a**) coated float glass with La−Si−O−N thin films (the dot on the backside is a reference for not coated side), (**b**) sapphire substrate coated with La−Si−O−N thin film and (**c**) bulk oxynitride glass prepared by melt-quenching technique.

For comparison purposes, thin films in the La−Si−O−N system were grown on soda-lime glass and sapphire substrates by RF magnetron sputtering apparatus. For the deposition, 50 mm diameter targets of lanthanum (purity 99.95%) and silicon (purity 99.99%) were used in an ultra-high vacuum (UHV) deposition system. For reactive sputtering, a mixture of Ar (31.4 sccm), nitrogen (8 sccm) and oxygen (0.6 sccm) was used, with a total gas flow of 40 sccm. The deposition time was 2 h. The compositional analysis by EDX and X-ray photoelectron spectroscopy (XPS) confirmed that obtained films contain a high amount of La and N. As shown in Figure 12, thin films in the La−Si−O−N system are optically transparent and free from metallic impurities as compared to bulk La−Si−O−N glass. A fair comparison of La containing thin films and bulk glasses is difficult due to differing synthesis techniques, stoichiometry, and dimensionality of the disordered network. In summary, it is possible to obtained optical transparent glasses with high nitrogen content by optimising the process parameters and the selection of the precursors.

## 4. Conclusions

The use of oxynitride glasses in optical applications depends on the minimisation or elimination of metallic silicides and free silicon defects that arise during synthesis. Metallic silicide and elemental silicon impurities were studied in nitrogen-rich La−Si−O−N glasses by optical, SEM and TEM microscopy. It was ascertained that these impurities are the main reason for the non-transparency of these glasses. The higher the amount of La silicides and free Si contents, the darker the glasses. The size of the La silicide was found to be below 1 µm, and its structure is amorphous or poorly crystalline. The elemental Si has a size of less than 100 nm. The quantities of elemental Si are much lower than the silicides. Nitrogen rich La−Si−O−N glasses show structural heterogeneities at the atomic or molecular scale. In contrast, nitrogen rich, thin films in the La−Si−O−N system are transparent and free from impurities. Furthermore, these thin films are smooth and homogenous. Impurities such as metal silicide and elemental silicon can be minimised by developing a low-temperature processing route, the use of AlN instead of Si_3_N_4_ as the source of nitrogen and use of high purity raw materials. However, much further work is needed to confirm (or possibly disprove) these statements.

## Data Availability

Not applicable.
